# Plant-derived extracellular nanovesicles: a promising biomedical approach for effective targeting of triple negative breast cancer cells

**DOI:** 10.3389/fbioe.2024.1390708

**Published:** 2024-06-17

**Authors:** Lishan Cui, Giordano Perini, Alberto Augello, Valentina Palmieri, Marco De Spirito, Massimiliano Papi

**Affiliations:** ^1^ Dipartimento di Neuroscienze, Università Cattolica del Sacro Cuore, Rome, Italy; ^2^ Fondazione Policlinico Universitario A. Gemelli IRCSS, Rome, Italy; ^3^ Istituto dei Sistemi Complessi, Consiglio nazionale delle ricerche (C.N.R.), Rome, Italy

**Keywords:** *Citrus limon L.*, extracellular nanovesicles, triple negative breast cancer, plant-derived extracellular vesicles, PI3K/AKT, MPAK/ERK

## Abstract

**Introduction:** Triple negative breast cancer (TNBC), a highly aggressive subtype accounting for 15–20% of all breast cancer cases, faces limited treatment options often accompanied by severe side effects. In recent years, natural extracellular nanovesicles derived from plants have emerged as promising candidates for cancer therapy, given their safety profile marked by non-immunogenicity and absence of inflammatory responses. Nevertheless, the potential anti-cancer effects of *Citrus limon* *L*.-derived extracellular nanovesicles (CLENs) for breast cancer treatment is still unexplored.

**Methods:** In this study, we investigated the anti-cancer effects of CLENs on two TNBC cell lines (4T1 and HCC-1806 cells) under growth conditions in 2D and 3D culture environments. The cellular uptake efficiency of CLENs and their internalization mechanism were evaluated in both cells using confocal microscopy. Thereafter, we assessed the effect of different concentrations of CLENs on cell viability over time using a dual approach of Calcein-AM PI live-dead assay and CellTiter-Glo bioluminescence assay. We also examined the influence of CLENs on the migratory and evasion abilities of TNBC cells through wound healing and 3D Matrigel drop evasion assays. Furthermore, Western blot analysis was employed to investigate the effects of CLENs on the phosphorylation levels of phosphoinositide 3-kinase (PI3K), protein kinase B (AKT), and extracellular signal- regulated kinase (ERK) expression.

**Results:** We found that CLENs were internalized by the cells via endocytosis, leading to decreased cell viability, in a dose- and time-dependent manner. Additionally, the migration and evasion abilities of TNBC cells were significantly inhibited under exposed to 40 and 80 μg/mL CLENs. Furthermore, down-regulated expression levels of phosphorylated phosphoinositide 3-kinase (PI3K), protein kinase B (AKT), and extracellular signal-regulated kinase (ERK), suggesting that the inhibition of cancer cell proliferation, migration, and evasion is driven by the inhibition of the PI3K/AKT and MAPK/ERK signaling pathways.

**Discussion:** Overall, our results demonstrate the anti-tumor efficiency of CLENs against TNBC cells, highlighting their potential as promising natural anti-cancer agents for clinical applications in cancer treatment.

## Introduction

Breast cancer is the most commonly diagnosed cancer and the leading cause of cancer-related deaths in women worldwide. Triple negative breast cancer (TNBC) accounts for approximately 15% of all breast cancer cases, which characterized by the absence of estrogen receptor, progesterone receptor, and HER2/neu expression (Marra et al., 2020). Patients with TNBC face significant clinical challenges as it is associated with poor prognosis, recurrence and distant metastasis, resulting in higher mortality rates in comparison to other subtypes of breast cancer (Bianchini et al., 2016). These challenges stem from the aggressive characteristics exhibited by TNBC and limited responsiveness to conventional chemotherapy treatments. With the notable advancements in drug screening technology, there is currently a surge in enthusiasm for developing new approaches to cancer treatment in the field of oncology. In particular, exosome-based therapy as one of the innovative strategies aim to address the challenge of drug resistance and reduce potential long-term complications associated with chemotherapy and radiotherapy (Yong et al., 2019; Xu et al., 2020; Luo et al., 2023).

Exosomes are small extracellular vesicles (EVs) with a diameter ranging from 30 to 150 nm, produced by prokaryotic and eukaryotic cells (Ambattu et al., 2020; Hosseini-Giv et al., 2022). These membrane vesicles play a key role in intercellular communication, facilitating the transfer of proteins, lipids, and nucleic acids between cells. This dynamic process is able to regulate cellular functions, thereby exerting a dual role of promoting or inhibiting the progression of cancer cells (Sun et al., 2018). On the one hand, tumor cell-derived exosomes are consistently implicated throughout all stages of cancer progression and play a critical role in therapy resistance (Li and Nabet, 2019; Wu et al., 2023). On the other hand, exosomes derived from immune cells have become key entities in the field of cancer therapy, given their immunomodulatory properties and therapeutic potential. The clinical trials conducted by Pitt et al. in patients with advanced malignancies highlighted the ability of dendritic cell-derived exosomes to activate T and NK cell-mediated immune responses, demonstrating their broad potential as immunotherapeutic agents (Pitt et al., 2016). Moreover, numerous studies have consistently demonstrated that exosomes derived from mesenchymal stem cells (MSCs) possess the capacity to effectively modulate the tumor microenvironment, alongside serving as potent carriers for tumor therapy (Fusheng et al., 2022; Lin et al., 2022; Sohrabi et al., 2022). There is evidence that engineered Exo-curcumin (CUR) + Indocyanine green (ICG) enhances the efficacy of chemo-phototherapy against glioma both *in vitro* and *in vivo* (Li et al., 2020). These exosomes successfully achieved dual drug loading and produced synergistic tumor treatment effects in solid tumor models (Wang et al., 2020). While mammalian cell-derived extracellular vesicles showed great promise in cancer treatment, challenges arise in scaling up production due to relatively low yields and specialized culture requirements. One of the main hurdles in utilizing these exosomes is the risk of immune rejection, which has the potential to limit their therapeutic efficacy by triggering undesired immune reactions (Jisu et al., 2022). Furthermore, there is a concern regarding the transfer of harmful genetic abnormalities, infections, or undesirable characteristics from donor cells to exosomes, potentially affecting the recipient (Robbins and Morelli, 2014; Buzas, 2023). Addressing these challenges is crucial to fully harness the potential of mammalian cell-derived exosomes for therapy.

In recent years, natural plant-derived exosome-like nanovesicles (PDENs) have been identified as emerging bioactive substances for cancer treatment. PDENs exhibit remarkable safety profiles, as they do not elicit immune system activation or induce inflammation. This characteristic renders them exceptionally well-suited for therapeutic use in human diseases, making them valuable for cell-free therapy. Furthermore, their relatively lower cost compared to other exosome sources establishes them as a more economically viable option for applications in biomedical settings. Thanks to their excellent biocompatibility and tumor-biasing properties, numerous extracellular vesicles extracted from edible plants and fruits, such as ginger (Zhang et al., 2016), lemon (Yang et al., 2020) and tea flowers (Qiubing et al., 2022) have been studied and exhibited significant anti-cancer properties against colitis-associated cancer, gastric cancer, and breast cancer, respectively. The nanovesicles derived from *Citrus limon L.* effectively suppressed cell growth in diverse cancer cell lines, including LAMA84 (human chronic myeloid leukemia cell line), SW480 (human colorectal adenocarcinoma cell line), and A549 (human lung carcinoma cell line), through the activation of TRAIL-mediated apoptosis (Raimondo et al., 2015).

In this study, CLENs with diameters of approximately 50–100 nm were obtained by ultracentrifugation and their physicochemical properties were characterized. CLENs internalized by endocytosis resulted in significant dose- and time-dependent inhibition of cell viability in both 4T1 and HCC-1806 TNBC cells. It is noteworthy that no cytotoxic effects were observed in normal Human Embryonic Kidney HEK-293 cells, highlighting that CLENs safety combined to anti-cancer properties, particularly towards TNBC cells. Moreover, the significant inhibition of cell migration and evasion by CLENs in 3D cancer models suggests that they have inhibitory effects on both tumor growth and metastasis processes. This inhibitory effect is mediated through inhibition of the PI3K/AKT and MAPK/ERK signaling pathways. Our findings shed light on the therapeutic potential of CLENs, bioactive nanovesicles derived from fruits, as a safe and effective treatment strategy for TNBC.

## Materials and methods

### Isolation and purification of nanovesicles from *Citrus limon L.*

*Citrus limon L.* purchased from a local market, carefully washed and squeezed. The juice was sequentially centrifuged at 3,000 × g for 30 min to remove dead cells, cell debris, and large particles. The supernatant was centrifuged at 10,000 × g for 60 min. Subsequently, the supernatant was filtered at 0.22 μm pore filter and centrifuged at 100,000 × g for 90 min in a fixed angle rotor Type 50.2 Ti (Beckman Coulter Inc., Brea, CA, USA). To further purify small EVs, the pellet was suspended in 5 mL cold PBS (1X) and transferred to a 30% sucrose/D_2_O cushion for ultracentrifugation. After centrifugation at 100,000 × g, 4°C for 120 min, the nanovesicle-containing fraction was resuspended in PBS (1X) and was centrifuged twice at 100,000 × g, 4°C for 90 min. The pellet was collected and resuspended in PBS (1X) for the subsequent experiments.

### Protein quantification by bicinchoninic acid (BCA) assay

The protein concentration of nanovesicles was determined using the BCA assay kit (Thermo Fisher Scientific, Waltham, MA), following the manufacturer’s instructions. Samples mixed with working reagent were incubated at 37°C for 30 min and then at room temperature for 20 min before being read. Protein concentration standard curve was obtained by using a series of bovine serum albumin (BSA) ranging from 25 μg/mL to 1,000 μg/mL. The absorbance was measured at 562 nm in a Cytation3 Cell Imaging Multi-Mode Reader (BioTek, Winooski, VT, USA).

### Transmission electron microscopy (TEM)

CLENs (10 μL) were added to Formvar/Carbon 200 mesh copper grids for 2 min at room temperature. The grids were dried using filter paper to remove excess moisture. After rinse with 10 μL of ddH_2_O, 10 μL of 5% uranyl acetate were dropped onto the copper grids. After 2 min, the excess staining solution was absorbed using filter paper, and the samples were air-dried for 30 min under the biological hood. The samples were observed by a Zeiss Libra 120 transmission electron microscope (TEM) at 80 kV.

### Dynamic light scattering (DLS)

Size distribution of CLENs was determined by dynamic light scattering (DLS) performed with a Zetasizer Nano ZS90 (Malvern Panalytical, Malvern, United Kingdom). Samples were diluted 1:100 with 0.22 μm filtered PBS prior to measurement. Average size distributions determined from triplicate measurements are reported. Data analysis was performed by Malvern Zetasizer software (Palmieri et al., 2014; Guglielmi et al., 2020).

### Nanoparticle tracking analysis (NTA)

The size distribution and concentration of CLENs were characterized by NanoSight NS300 (Malvern Technologies, Malvern, United Kingdom). Accurate nanoparticle tracking was verified using a 30 nm gold nanoparticle standard (Sigma Aldrich, product #753629, St. Louis, MO, USA) prior to examination of the samples. CLENs were diluted 100-fold with sterile filtered (0.22 μm pore size) PBS for NTA measurement. Five videos of each sample were captured with a high-resolution camera to obtain the mean and standard error, and outcomes were analyzed with NTA 3.4 software.

### Cell culture

The human embryonic kidney-293 (HEK-293) and murine mammary tumor cell line 4T1 were maintained in culture in Dulbecco’s Modified Essential Medium (DMEM, Sigma-Aldrich, St. Louis, MO, USA) enriched with 10% fetal bovine serum (FBS, Gibco, Life Technologies) and 2% penicillin–streptomycin (Sigma-Aldrich, St. Louis, MO, USA). Human basal-like TNBC cell line HCC-1806 was maintained in culture in Roswell Park Memorial Institute (RPMI) 1,640 medium (Thermo Fisher Scientific, Waltham, MA, USA) llemented with 10% fetal bovine serum (FBS) and 2% penicillin–streptomycin. The cells were purchased from American Type Culture Collection (ATCC, Rockville, MD, USA) and maintained at 37°C with 5% CO_2_ under a humidified atmosphere.

### Cellular uptake by confocal microscopy

The CLENs (100 μL) isolated as described above were stained with 50 μM Calcein-AM for 30 min at 37°C. After incubation, the labeled CLENs were resuspended in 5 mL of 0.22 μm filtered PBS and subjected to ultracentrifugation at 100,000 x g for 60 min. The pellet was carefully resuspended in 100 μL PBS, ready for subsequent incubation with cells. 4T1 and HCC-1806 cells were seeded at a density of 1 × 10^4^ cells/cm^2^ per well in an 8-well chambered coverglass (Cellvis, Cat. #: C8-1.5-H-N). The day after, cells were incubated with 20 μg/mL of labeled-nanovesicles for 4 h at 37°C or 4°C. After labelling, the cells were fixed with 4% paraformaldehyde for 20 min at room temperature, and then washed two times with PBS. Fixed cells were permeabilized with 0.1% Triton^™^ X-100 (Sigma-Aldrich) for 15 min at RT, followed by two washes with PBS. Subsequently, cells were stained with one unit of rhodamine phalloidin (Life Technologies, Molecular. Probes) for 1 h at RT, and the nucleus was stained with DAPI for 15 min in the dark condition at room temperature. Confocal microscopy was used to visualize the cellular uptake of CLENs after 4 h of incubation. Fluorescence images were acquired using Nikon’s A1 MP + multiphoton confocal microscope, equipped with a ×60 oil immersion objective.

### CellTiter-Glo luminescent cell viability assay

Cell viability of HEK-293, 4T1 and HCC-1806 cells were evaluated by CellTiter-Glo^®^ Luminescent Cell Viability Assay (Promega, Madison, WI, USA). The 1 × 10^5^ cells were seeded in each well of the 96-well plate in a complete DMEM medium and incubated overnight at 37°C with 5% CO_2_. After 24 h, cells were treated with CLENs at serial concentrations of 5, 10, 20, 40 and 80 μg/mL in DMEM supplemented with 2% FBS, as low serum conditions can reduce the availability of growth factors and nutrients necessary for cell survival. After incubating for 24, 48, or 72 h at 37°C, the cells were thoroughly washed with PBS to remove excess CLENs, and then equal volume of CellTiter-Glo^®^ reagent were added to the cell culture medium present in each well and incubated for 2 min with an orbital shaker to induce cell lysis. The plate was incubated for an additional 10 min at room temperature in the dark to stabilize the luminescence signal. After incubation, luminescence was measured by Cytation 3 Cell Imaging Multi-Mode Reader (BioTek, Winooski, VT, USA).

### Live/dead assay by calcein AM − PI

Live/dead staining was performed using Calcein AM and propidium iodide (PI). Calcein-AM itself is a non-fluorescent analog of calcein, but the highly lipophilic acetoxymethyl ester of calcein permeates cell membranes, and intracellular esterase generate calcein from Calcein-AM, which emits intense green fluorescence. Calcein AM, a cell-permeable dye that stains only living cells, whereas PI is a cell-impermeable DNA-binding dye that penetrates the cell membrane of dead or dying cells. The 1 × 10^5^ of HEK-293, 4T1 and HCC-1806 cells were seeded in the 96-well plate in a complete DMEM medium and incubated overnight at 37°C with 5% CO_2_. The cells were treated with CLENs at concentrations of 5, 10, 20, 40, and 80 μg/mL for 24, 48, and 72 h. Subsequently, the cells were incubated with green-fluorescent Calcein-AM for 20 min, followed by a 5 min co-culture with PI. The fluorescence images were observed by Cytation 3 Cell Imaging Multi-Mode Reader (BioTek, Winooski, VT, USA), with excitation at 488 nm and emission recorded at 525 nm.

### Wound healing assay

A density of 5 × 10^5^ cells was seeded in a 12-well plate and incubated overnight at 37°C in a 5% CO2 incubator. The day after, when the cells reached density of 90% confluence, the cell monolayers were scratched using a sterile P-200 pipette tip and washed twice with PBS to remove the floating cells and debris. The PBS was pre-warmed at 37°C to avoid detaching cells during the wash. Subsequently, the cells were treated with low serum (2% FBS) medium containing CLENs at concentrations of 40 and 80 μg/mL or without treatment. Wound healing images were continuously captured at indicated time points using Cytation 3 Cell Imaging Multi-Mode Reader (BioTek, Winooski, VT, USA) with ×4 magnification. The remaining wound area was measured using ImageJ software.

### Matrigel drops evasion assay

The cells were trypsinized and resuspended in cold serum free DMEM mixed with cold Matrigel (Corning; catalog no.: 354,234) at a final concentration of 8 mg/mL. Four drops of Matrigel containing 7,500 cells per drop were plated in six well plates by BioX (Cellink) 3D-bioprinter. All materials were pre-chilled and experiments were performed on ice to avoid gelation. The drops were allowed to solidify by upside-down the plate and placing it in a humidified incubator at 37°C, under 5% CO_2_ for 30 min. Afterwards, the plate was inverted again and left for an additional 30 min, and then 3 mL of complete medium is added to each well. Images were taken at day 0, day 7, and day 10 post-incubation, both in the absence and presence of CLENs. Quantification of cell aggregates and their respective areas were analyzed using ImageJ software (Moriconi et al., 2017; Perini et al., 2022).

### Western blot analysis

After 72 h of incubation, cellular protein extraction was performed by homogenizing cells in cell lysis buffer (#FNN0011, Invitrogen) supplemented with an EDTA-free Protease Inhibitor (Thermo Scientific™) and PMSF Protease Inhibitor (Thermo Scientific™). Subsequently, the resulting homogenates were subjected to Western blot analysis using established protocols, as previously described (Cui et al., 2022). Briefly, a total of 20 µg of proteins extracted from cell lysates were loaded onto a 4%–20% Mini-PROTEAN^®^ TGX^™^ Precast Protein Gel (Bio-Rad, Hercules, CA, USA), and transferred to a polyvinylidene difluoride (PVDF) transfer membrane (Thermo Scientific^™^). The membrane was blocked by EveryBlot Blocking Buffer (Bio-Rad, Hercules, CA, USA) for 10 min to prevent non-specific binding. Subsequently, the membrane was incubated overnight at 4 °C with primary antibodies (PI3K #4257, p-PI3K #17366, Akt #9272, p-Akt #9271, Erk #4695, p-Erk #4370 and β-actin #93473, from Cell Signaling Technology, with a dilution ratio of 1:1,000). The membranes were washed three times with TBS-T and then incubated with secondary antibody (Anti-rabbit IgG, HRP-linked Antibody #7074, from Cell Signaling Technology, 1:2000) at room temperature for 1 h. After three washes with TBS-T, the protein bands were incubated with Pierce^™^ ECL Western blotting Substrate (Thermo Scientific^™^) and detected using the Alliance Q9 Advanced System (UVITEC, Cambridge, United Kingdom). Densitometric analysis was performed by using ImageJ software.

### Statistical analysis

Quantitative data were obtained from three independent experiments and are presented as means ± SEM. Statistical analysis was performed using one-way ANOVA and two-way ANOVA, followed by Tukey’s multiple comparison *post hoc* test, to determine the significance of differences. Statistical analysis was executed with GraphPad Prism9 software (San Diego, CA, USA), using *p* < 0.05 as the critical level of significance (*p < 0.05; **p < 0.01; ***p < 0.001; ****p < 0.0001). Three independent experiments done in triplicate.

## Results

### Isolation and characterization of extracellular nanovesicles from *Citrus limon L.*

**FIGURE 1:**
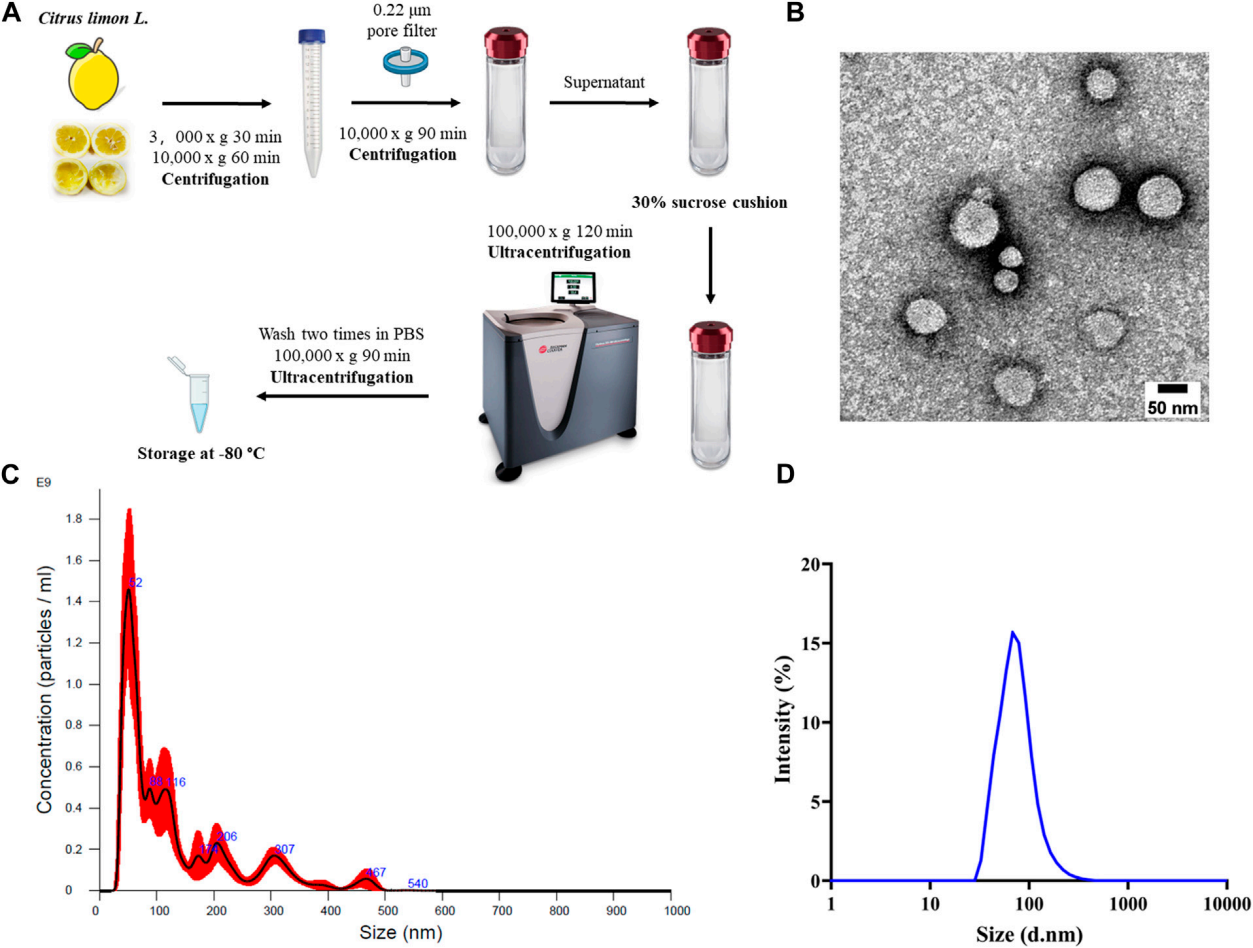
Isolation and characterization of *Citrus limon L.*-derived nanovesicles. **(A)** Schematic diagram of the working principle of 30% sucrose-based ultracentrifugation for isolation and purification of CLENs. **(B)** Representative TEM image of CLENs (scale bar = 50 nm). **(C)** Size distribution and concentration of CLENs determined by the nanoparticle tracking analysis (NTA). **(D)** Size distribution of CLENs detected by dynamic light scattering (DLS).

One-step sucrose cushion ultracentrifugation approach (Figure 1A) was employed to extract extracellular nanovesicles from *C. limon L.* juice. This methodology is widely regarded as the “gold standard” for EV isolation due to its established effectiveness and reliability (Gupta et al., 2018). A representative TEM image was reported in Figure 1B shows that CLENs are spherically shaped with a diameter of approximately 50–100 nm, which is consistent with the results obtained from dynamic light scattering (DLS) analysis (Figure 1C) and Nanoparticle Tracking Analysis (NTA) measurements (Figure 1D), ensured unambiguous determination of the nanovesicle size, thus strengthening the reliability of the findings. Furthermore, the NTA revealed a concentration of the nanovesicles to be 1.03 e+11 ± 1.71 e+10 particles/mL.

### Cellular uptake of CLENs by triple-negative breast cancer cells

**FIGURE 2:**
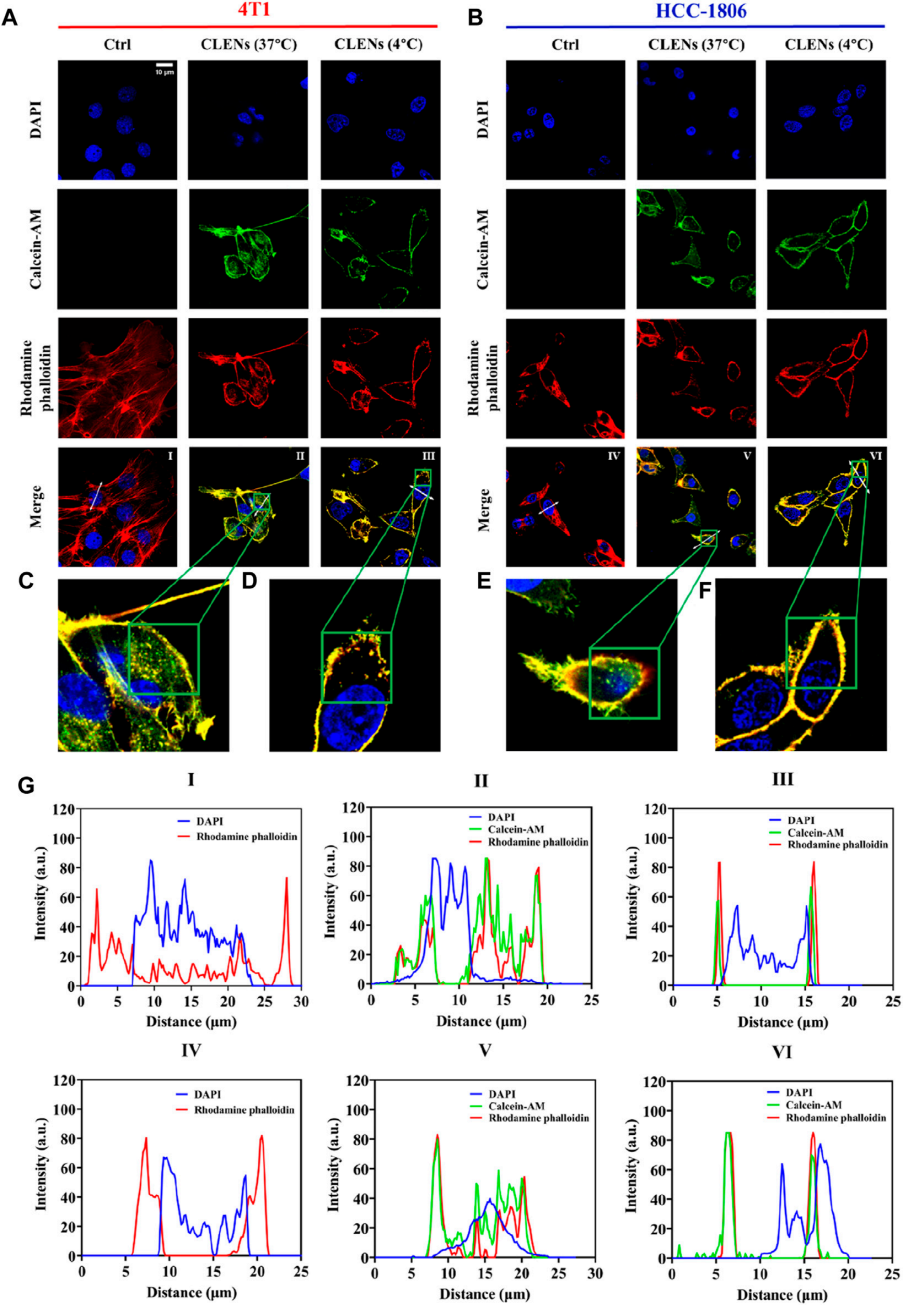
Cellular uptake of CLENs in TNBC cells. Confocal microscopy analysis of the uptake of green fluorescently labeled CLENs in both 4T1 **(A)** and HCC-1806 **(B)** followed by incubation for 4 h at 37°C and 4°C, respectively. The actin filaments were labeled with rhodamine phalloidin (shown in red), while the cell nuclei were counterstained with 4′,6-diamidino-2-phenylindole (DAPI) (scale bar = 10 µm). The magnified images depict high-magnification views of the boxed region **(C–F)**. The fluorescence profiles of 4T1 and HCC-1806 cells plotted along the white arrows were analyzed by ImageJ software **(G)**.

The cellular uptake of nanovesicles offers valuable insights into their potential therapeutic response, as a high uptake specifically by cancer cells implies the potential for enhanced treatment outcomes. The human HCC-1806 cells and the mouse 4T1 cells were used as representative models of TNBC. The uptake behavior of CLENs in 4T1 and HCC-1806 TNBC cells at 37°C and 4°C was obtained by confocal microscopy, allowing a thorough investigation of its internalization pattern. As shown in Figures 2A, B, following 4 h of incubation at 37°C, the extracellular nanovesicles was co-localized with intracellular vesicular structures, highlighting the successful uptake by both cell lines. A large number of labeled nanovesicles can be observed within the cytoplasm (Figures 2C, E). In general, the nanovesicles uptake occurs via energy-dependent (ED) mechanisms, energy-independent (EI) mechanisms, or a combination of both. Endocytosis is a well-studied ED pathway for the transportation of nanoparticles across the cellular membrane, facilitating their internalization into specific cells and subsequent accumulation, which includes phagocytosis, macropinocytosis, clathrin- or caveolin-mediated endocytosis. Conversely, EI internalization mechanisms involve fusion, embedment, and direct translocation (Quagliarini et al., 2022). Traditional small molecular drugs primarily enter cells through passive or active process, whereas nanomedicines enter cells via endocytosis (Longfa et al., 2013). To evaluate the uptake process, we exploited temperature inhibition. Results in Figures 2D, F show that the uptake was inhibited at lower temperatures (4°C) in both cell lines. The fluorescence profiles of 4T1 and HCC-1806 cells are shown in Figure 2G. Notably, the majority of labeled-nanovesicles are intercepted in the cell membrane, providing evidence to suggest that the uptake likely occurred through endocytosis, facilitates efficient interactions of CLENs with recipient cells, resulting in profound biological effects.

### CLENs possess specific anti-cancer properties targeting TNBC cells

**FIGURE 3:**
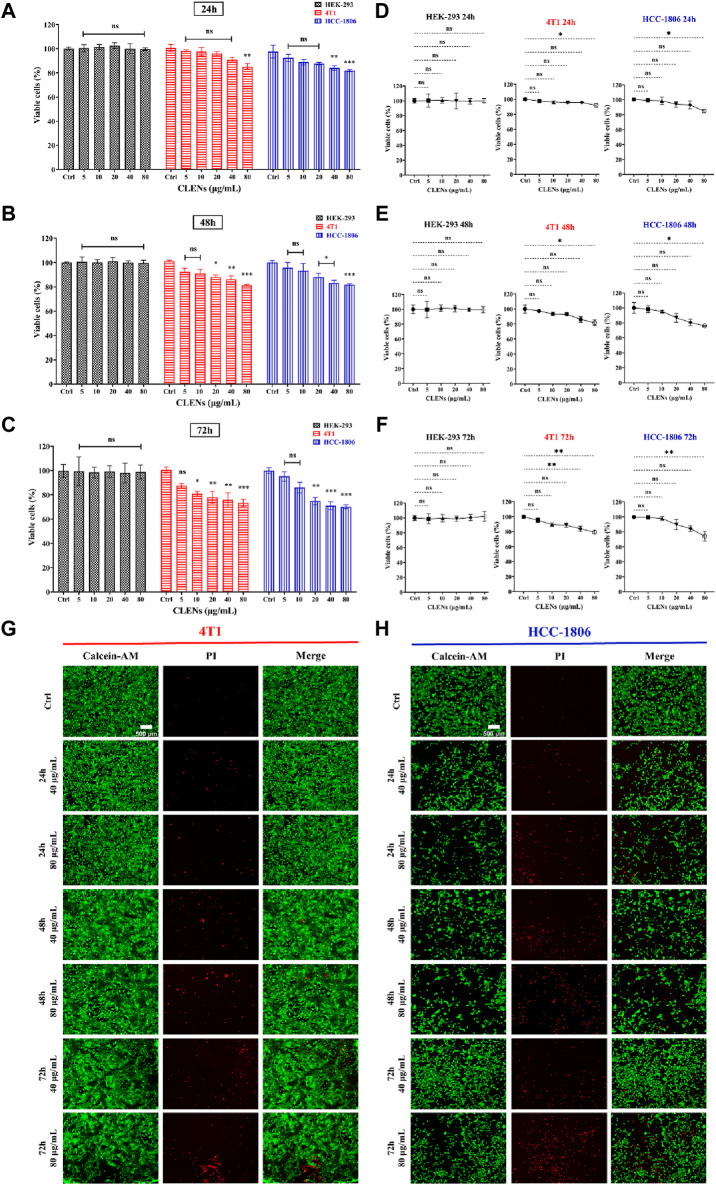
Effect of CLENs on the cell viability of HEK-293, 4T1 and HCC-1806 cells. Cells were incubated with or without treatment with increasing concentrations of CLENs for 24 h **(A)**, 48 h **(B)** or 72 h **(C)** and cell viability was determined by CellTiter-Glo assay. Calcein-PI live-death assay quantifies live and dead cell populations as a line graph showing the effect of different concentrations of CLENs at 24 h **(D)**, 48 h **(E)**, and 72 h **(F)**. The results indicate the proportion of living cells compared to the cells that were not treated (untreated control cells). Representative fluorescence images of 4T1 **(G)** and HCC-1806 **(H)** cells received 40 and 80 μg/mL CLENs for 24, 48, and 72 h (scale bar = 500 μm). Columns, mean of three independent experiments performed in sextuplicate; Bars, Mean ± SEM. Statistical significance was evaluated using two-way ANOVA and one-way ANOVA followed by Tukey’s *post hoc* tests (*p < 0.05; **p < 0.01; ***p < 0.001).

We used the human cell line HEK-293 as a model to determine the specificity of nanovesicles towards TNBC cells compared to non-aggressive cell lines. The cells were incubated with indicated concentrations of CLENs for 24h, 48h and 72 h prior to bioluminescent assay (Figures 3A–C). CLENs exhibited a significant inhibition of cell viability in both TNBC cell lines, and the magnitude of the effect was time- and dose-dependent. In particular, the administration of 80 μg/mL of CLENs to 4T1 cells resulted in a cell viability of 85.1% and 81.2% after 24 and 48 h of incubation, respectively. Notably, upon extending the incubation period to 72 h, a substantial reduction of 73.4% in cell viability was observed. A similar trend was observed in the HCC-1806 cell line, wherein a 24-h incubation with the highest concentration of CLENs resulted in a cell viability of 81.9%, which slightly decreased to 81.8% after 48 h. However, the viability dropped further to 70.3% after 72 h of incubation. It is worth noting that no cytotoxicity was observed in HEK-293 cells after CLENs administration, highlighting the specific selectivity of CLEN for TNBC cells. Furthermore, to validate the results obtained from the bioluminescent assay, we conducted an additional Calcein-PI live-dead assay (Figures 3D–F). Notably, the administration of CLENs at the highest concentration (80 μg/mL) to 4T1 cells led to successive decreases in cell viability of 8.1%, 18.3%, and 20.8% after 24, 48, and 72 h of incubation, respectively. In the case of HCC-1806 cells, there was a decrease of 14.9%, 24.2%, and 25.9% following the respective incubation periods. Figures 3G, H illustrate the most representative images obtained from the Cal-PI live-dead assay after incubating with 40 and 80 μg/mL CLENs for 24, 48, and 72 h. The Supplementary Figure S3 provides images of live and dead cells showing the effect of administering different concentrations of CLENs. The results obtained from the CellTiter-Glo assay in line with the findings from the Cal-PI live-dead assay, suggest that CLENs have potential anticancer effects by inhibiting tumor cell proliferation. Notably, no cytotoxic effects were observed in normal Human cells HEK-293 cell line, implying CLENs themself possess anti-cancer properties specifically towards TNBC cells.

### CLENs effectively inhibit the migration and evasion of TNBC cells

**FIGURE 4:**
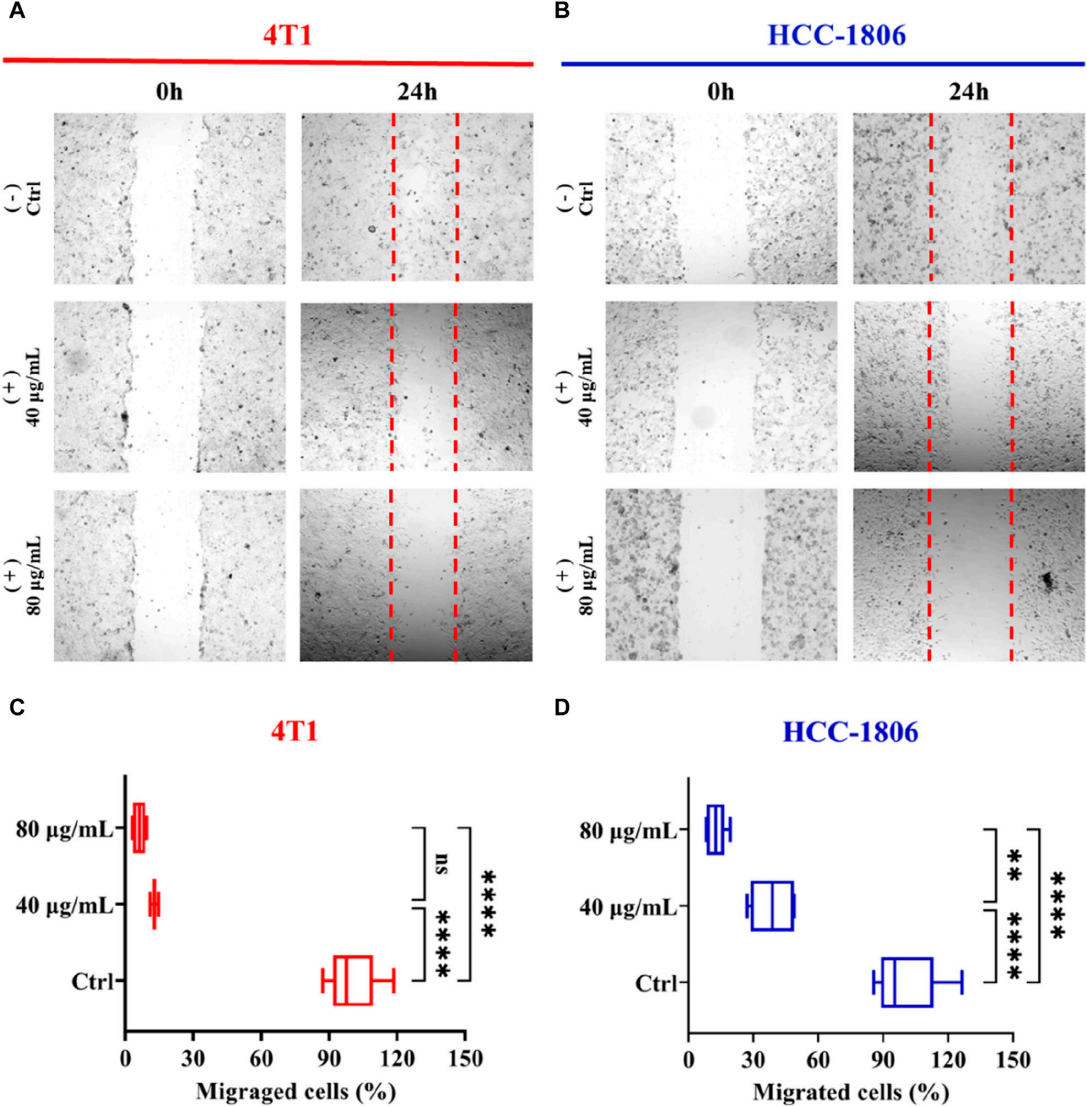
Effects of CLENs on 4T1 and HCC-1806 cells migration. Wound healing assay performed in 4T1 **(A)** and HCC-1806 **(B)** cells scratched to cause a wound were incubated without or with 40 and 80 μg/mL CLENs. Representative images are shown the cell migration into wound area 24 h post-wounding. Graphical representation **(C,D)** of data from three independent experiments performed in triplicate. Results are expressed as percentages taking into account the ratio of migrated cells to control cells. Bars, Mean ± SEM. *p < 0.05; **p < 0.01; One-way ANOVA followed by Tukey’s multiple comparisons test.

To elucidate the inhibitory effect of CLENs on the migratory behavior of 4T1 and HCC-1806 cells, we conducted a wound-healing assay. The results revealed a significant finding regarding the impact of CLENs on cell migration in TNBC cell lines. Following a 24-h incubation period in the absence or presence of 40 and 80 μg/mL of CLENs, a significant and dose-dependent inhibitory effect of CLENs on the migratory behavior of both 4T1 and HCC-1806 cell lines was observed (Figures 4A, B). Quantitative analysis was performed to assess the migratory cell population that infiltrated the wound area. Notably, upon exposure to CLENs at concentrations of 40 and 80 μg/mL, the migratory potential of the 4T1 cell line exhibited a substantial reduction of −87.1% and −93.8% respectively (Figure 4C). Equally remarkable, the HCC-1806 cell line demonstrated a pronounced inhibition of migration by −61.2% and −87.5% with the corresponding CLENs concentrations (Figure 4D). It is noteworthy that in human TNBC HCC-1806 cell line, the inhibitory effects reached 40.8% and 75.8% respectively, and this inhibitory effect persisted even after a 48-h. Furthermore, the inhibitory effects demonstrated remarkable persistence, as evidenced by the observation of 37.2% and 72.9% inhibition after a 72-h, indicating a prolonged and sustained impact on the migratory behavior of the cells (Supplementary Figure S3).

**FIGURE 5:**
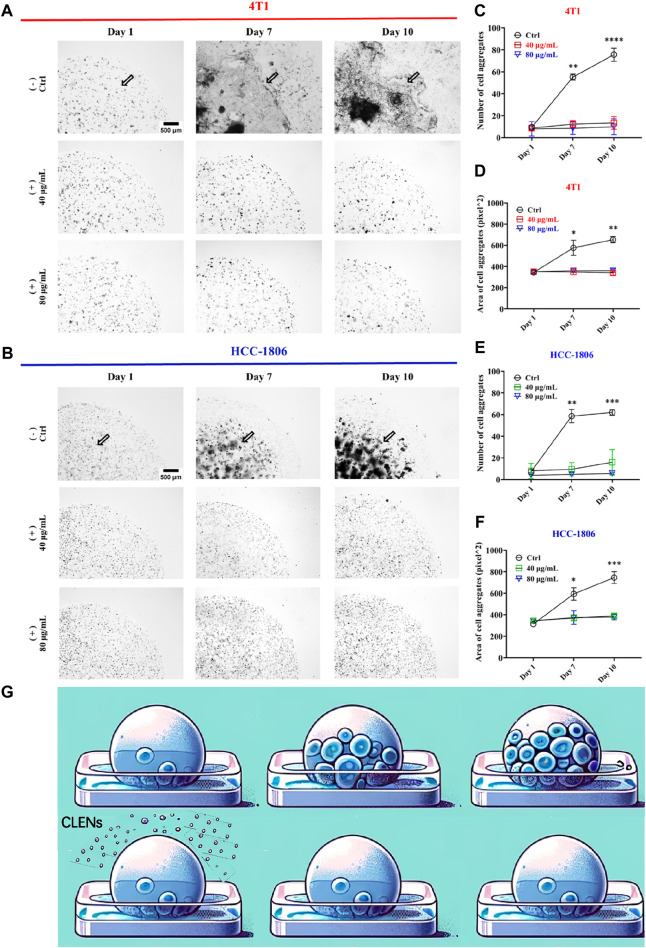
CLENs effectively prevents the evasion of 4T1 and HCC-1806 cells. Representative images of 4T1 **(A)** and HCC-1806 **(B)** cells were acquired at day 0, day 7 and after 10 days of incubation in the absence or presence of CLENs at concentrations of 40 and 80 μg/mL (scale bar = 500 μm). Graphical representation of the number of cell aggregates **(C,E)** and the area of aggregated cells **(D,F)** in 4T1 and HCC-1806 cells were shown. An illustrative representation of the experiment is depicted, where the addition of CLENs inhibits cell proliferation within the polymeric microenvironment of the bioprinted spheroid **(G)**. Data from three independent experiments performed in triplicate were analyzed by ImageJ software. Statistical significance was evaluated using two-way ANOVA followed by Tukey’s *post hoc* tests (*p < 0.05; **p < 0.01; ***p < 0.001).

Next, we conducted a Matrigel drop evasion assay to assess the evasion and migration capability of TNBC cells. 3D structured Matrigel droplets prepared with 4T1 and HCC-1806 cells were incubated for 10 days in culture medium supplemented with or without CLENs. Notably, exposure to the presence of CLENs resulted in a significant inhibition of cell migration in both cell lines. In contrast, invadopodia formation was observed in the control group of cells, representing specialized protrusions that exert a pivotal role in facilitating the dissemination of cancer cells. In detail, the aggressive growth of 4T1 cells was distinctly observed after 7 and 10 days in the absence of CLENs, whereas the presence of 40 and 80 μg/mL CLENs effectively regulated cell evasion (Figure 5A). Migrated cell counts showed a significant increase, with levels in the control group soaring 5-fold and 7-fold relative to CLENs treatment on day 7 and 10, as shown in Figure 5C. Moreover, the control group showed cell aggregation areas that were 1.5 and 2 times larger than those observed in the groups treated up to day 7 and day 10, respectively (Figure 5D). Interestingly, we noticed that HCC-1806 cells have a unique proliferative behavior within 3D Matrigel droplets that differs from patterns observed in 4T1 cells under same conditions. The control group displayed a notable tendency of cell aggregation and strong intercellular interactions in the absence of CLENs. Conversely, the presence of CLENs effectively suppressed the formation of cell aggregates in HCC-1806 cells (Figure 5B). By days 7 and 10, the number of aggregated cells in the control group increased 5- and 6-fold compared to those treated with CLENs (Figure 5E). In terms of aggregate formation area, on days 7 and 10, the inhibitory effects of 40 μg/mL and 80 μg/mL CLENs on HCC-1806 cells were 1 and 2 times that of the control group, respectively (Figure 5F). A schematic diagram is shown in Figure 5G. Our findings provided compelling evidence supporting the inhibitory effects of CLENs on the metastatic potential of TNBC cells.

### The effects of CLENs on PI3K/AKT and MAPK signaling pathways

**FIGURE 6:**
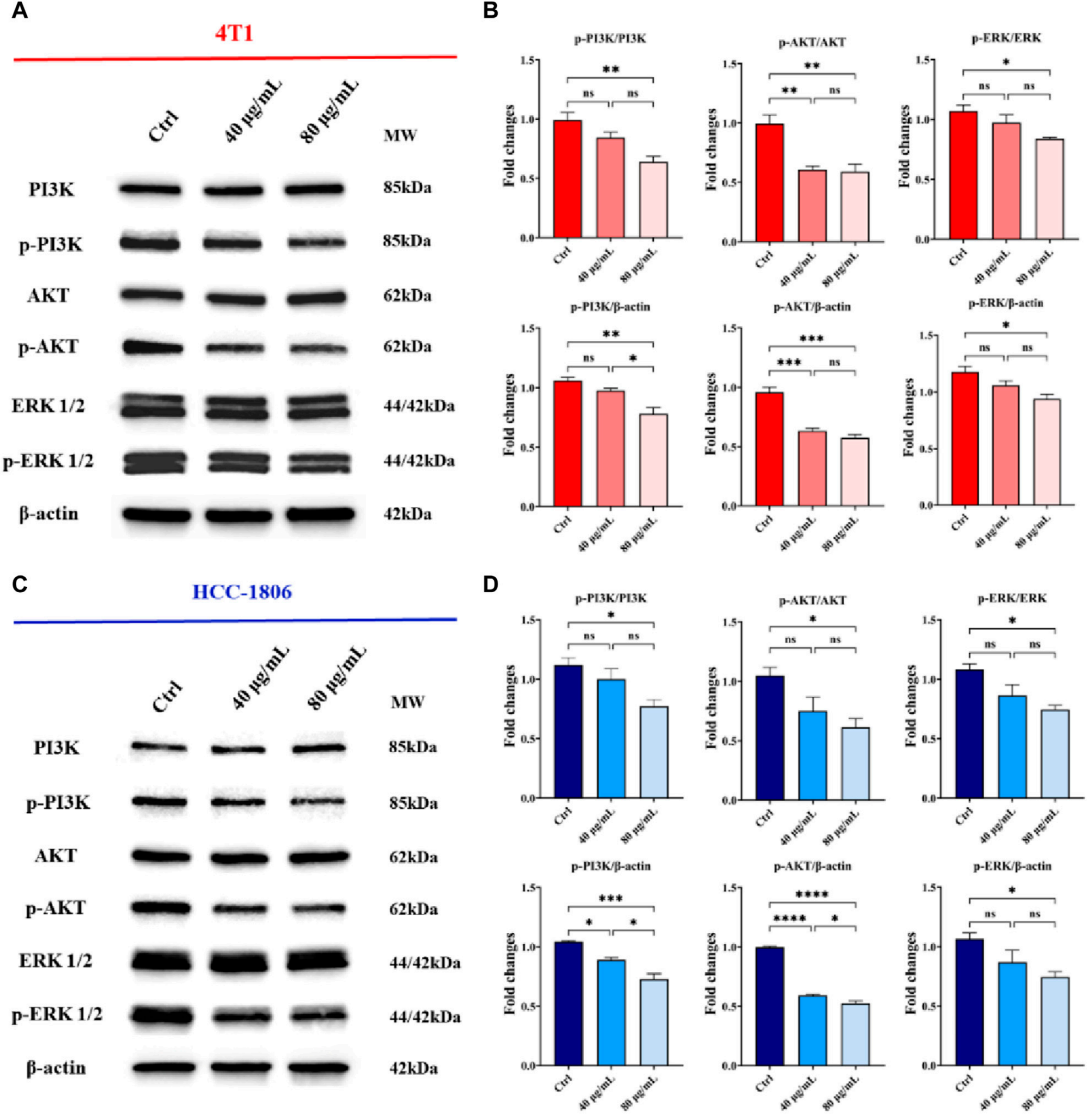
CLENs treatment resulted in a downregulation in the expression of phosphorylated forms of PI3K, AKT, and ERK in TNBC cells. Representative Western blot analysis of PI3K/AKT and MAPK/ERK signaling pathways in **(A)** 4T1 and **(C)** HCC-1806 cells, untreated (Ctrl) or treated with 40 μg/mL and 80 μg/mL, respectively. β-Actin was used as a loading control. Equal amounts of protein (20 mg) were loaded. **(B,D)** Densitometric analysis of p-PI3K, p-AKT, and p-ERK expression, normalized on β-actin, and of p-PI3K/PI3K, p-AKT/AKT and p-ERK/ERK. Data obtained from three independent experiments. One-way ANOVA test followed by Tukey’s multiple comparison test (*p < 0.05; **p < 0.01; ***p < 0.001).

The phosphatidylinositol 3-kinase (PI3K)/protein kinase B (AKT) and mitogen-activated protein kinase (MAPK)/extracellular signal-regulated kinase (ERK) signaling pathways are the key signaling pathways involved in the regulation of multiple cellular physiological processes by activating downstream corresponding effector molecules, which serve an important role in the cell cycle, growth and proliferation that mediate cancer cell survival and proliferation (Pascual and Turner, 2019). To delve into the molecular mechanisms underlying the anticancer effects of CLENs, we performed Western blot analysis on 4T1 and HCC-1806 cells, both untreated and treated with varying concentrations (40 μg/mL and 80 μg/mL) of CLENs over a 72-h period. Specifically, we examined the levels of total and phosphorylated forms of PI3K, AKT, and ERK. The representative images were shown in Figures 6A, C, with a dose-dependent downregulation of phosphorylated PI3K (p-PI3K), phosphorylated AKT (p-AKT), and ERK (p-ERK) in both 4T1 and HCC-1806 cells following exposure to 40 and 80 μg/mL CLENs (Figures 6B, D). Collectively, our findings suggest that CLENs exert suppressive effects on the PI3K/AKT and MAPK/ERK signaling pathways in both 4T1 and HCC-1806 TNBC cells.

## Discussions

Recently, substantial focus has been directed towards the exploration and identification of novel anticancer compounds derived from natural origins. The utilization of extracellular vesicles derived from edible plants and fruits has garnered significant attention as a novel anti-tumor therapy strategy (Nemati et al., 2022; Sarasati et al., 2023), thanks to their natural characterization of superior ability to bypass immune system activation and reduce inflammation (Jisu et al., 2022). However, so far there is a lack of studies reporting the anti-cancer effects of CLENs on TNBC, and the mechanism underlying carcinogenesis suppression remains an ongoing effort. In the present study, we characterized CLENs from *Citrus Limon L.* juice and evaluated their anti-cancer effects on both 4T1 and HCC-1806 TNBC cells.

The ultracentrifugation is crucial for preserving the structural integrity of EVs, as any disruption to EVs can potentially impair their biological functions, therefore, to extract high-quality CLENs from *Citrus Limon L.* juice, we employed a sucrose-based one-step ultracentrifugation method. CLENs characterization revealed visual evidence of spherical nanovesicle structures in intact form, with a diameter distribution ranging from approximately 50–100 nm, similar to mammalian cell derived exosomes (Kakarla et al., 2020; Zhang and Cheng, 2023). In the last decade, numerous therapeutic investigations have been conducted using exosomes derived from mammalian cells. Among them, mesenchymal stem cell-derived exosomes have emerged as particularly noteworthy due to their demonstrated capacity for self-renewal and multilineage differentiation potential (Sarugaser et al., 2009). Nonetheless, challenges remain in terms of relatively low manufacturing yields, difficulties in *in vitro* amplification, and authorization for clinical applications (Tang et al., 2021). Consequently, attention has shifted towards natural plant sources as alternatives to address the limitations associated with mammalian-derived exosomes. The utilization of plant-derived exosomes is particularly appealing due to their cost-effectiveness, biocompatibility, intrinsic anti-tumor properties, and the feasibility of industrial-scale manufacturing (Alzahrani et al., 2023; Chen et al., 2023; Xu et al., 2023). These features make them as emerging nanomedical agents of great interest in therapeutic research across multiple cancer types (Guglielmi et al., 2020; Stanly et al., 2020; Özkan et al., 2021; Chen et al., 2022). The exosomes derived from Citrus fruits have shown anti-proliferative activity in various types of cancer cell line (Kawaii et al., 1999; Cirmi et al., 2016). Especially, *C. limon L.*-derived exosomes harvested promising anti-cancer effects in colorectal cancer cells (Takakura et al., 2022) and gastric cancer cells (Yang et al., 2020) *in vitro* and even in CML xenograft mice models (Raimondo et al., 2015).

In order to determine the anti-proliferation effects of CLENs on TNBC, we selected two cancer cell lines, 4T1 and HCC-1806, and compared to human cell line HEK-293. Given the natural intrinsic tumor-targeting properties, we anticipated that CLENs would not induce cytotoxicity in the HEK-293 cell line. Indeed, our results showed that CLENs tend to selectively target tumor cells while maintaining the viability of non-malignant human cell lines. The observed decline in cell proliferation among TNBC cells in response to CLENs treatment may be attributed to the cellular uptake of these nanovesicles. The internalization of nanovesicles by cancer cells represents a key factor that leads to the reduction in cancer cell proliferation and adhesion, as it inhibits the metastatic spread of cancer cells to their target organs (Yin et al., 2014). Hence, we examined the cellular uptake efficiency of CLENs in both 4T1 and HCC-1806 TNBC cancer cells. Our observations indicate that CLENs were internalized through the process of endocytosis. Relatively higher rates of recurrence and distant metastasis of TNBC with respect to other subtypes of BC, remains huge challenge in developing effective cancer therapies (Bianchini et al., 2016; Zagami and Carey, 2022). Nevertheless, CLENs showed an excellent ability to significantly inhibit the migration and invasion propensity of both TNBC cell lines. Notably, these effects were more pronounced for HCC-1806 cells (Supplementary S3). Inhibition of cancer cell invasion and migration capabilities is strongly correlated with inhibition of the PI3K/AKT signaling pathway (Li et al., 2017; Jiang et al., 2018). The PI3K/AKT and MAPK/ERK signaling pathways stand out as essential signaling pathways across various types of cancer (He et al., 2021; Moon and Ro, 2021), with particular significance as key contributors in chemoresistance and survival of TNBC (Bartholomeusz et al., 2012; Costa et al., 2018; Nedeljković and Damjanović, 2019). Stanly et al. observed *Citrus paradisi*-derived nanovesicles suppress phosphorylation of ERK and AKT in melanoma A375 cells (Stanly et al., 2020). It was also reported that *Momordica.charantia*-derived extracellular vesicles-like nanovesicles (MCELNs) resulted in downregulation of p-PI3K and p-AKT in U251 glioma cells. Results displayed in Figure 5 were in line with these results, the cells treatment with CLENs displayed a statistically significant downregulated the expression of phosphorylated PI3K, AKT and ERK. Based on the findings, we suggest that CLENs inhibit cancer cell proliferation, migration and evasion through their inhibitory effects on the PI3K/AKT and MAPK/ERK signaling pathways.

## Conclusion

We implemented a highly efficient protocol for the isolation of extracellular vesicles from *C. limon L.* utilizing one-step sucrose cushion ultracentrifugation, resulting in the acquisition of CLENs with high quality and yields. The obtained CLENs exert anti-cancer properties on TNBC cells by inhibiting cell proliferation, migration and evasion through inhibition of both PI3K/AKT and MAPK/ERK signaling pathways. This effect was consistently observed in both 2D and 3D models, including within a polymeric matrix simulating a microenvironment conducive to cell growth and the formation of new metastases. These results highlight the potential of CLENs as a complementary therapy in combination with conventional oncological pharmacotherapies to mitigate the spread of circulating tumor cells and enhance treatment efficacy. The biocompatible and biosafe characteristics of CLENs provides valuable insights into the therapeutic potential of plant-derived extracellular vesicles. As an emerging natural nanomedicine, CLENs holds great promise for enhancing cancer therapy and improving patient outcomes in the field of cancer treatment.

## Data Availability

The original contributions presented in the study are included in the article/Supplementary Material, further inquiries can be directed to the corresponding authors.

## Abbreviations

AKT, Phosphorylated protein kinase B; BSA, Bovine serum albumin; Cal, Calcein-AM; CLENs, *C. limon* L.-derived extracellular nanovesicles; DLS, Dynamic light scattering; DMEM, Dulbecco’s Modified Essential Medium; ED, Energy-dependent; EI, Energy-independent; ERK, Extracellular signal-regulated kinase; EVs, Extracellular vesicles; FBS, Fetal bovine serum; HEK-293, Human embryonic kidney-293; MSCs, Mesenchymal stem cells; NTA, Nanoparticle tracking analysis; PDENs, Plant-derived exosome-like nanovesicles; PI3K, Phosphoinositide 3-kinase; PI, Propidium iodide; PVDF, Polyvinylidene difluoride; p-AKT, Phosphorylated AKT; p-ERK, Phosphorylated ERK; p-PI3K, Phosphorylated PI3K; RPMI, Roswell Park Memorial Institute; TEM, Transmission electron microscopy; TNBC, Triple negative breast cancer.
