# Supplementary material for: Plant-derived extracellular nanovesicles: a promising biomedical approach for effective targeting of triple negative breast cancer cells
Source: Front Bioeng Biotechnol. 2024 Jun 17;12:1390708. doi: 10.3389/fbioe.2024.1390708 (PMC11215178; doi:10.3389/fbioe.2024.1390708)
Supplement: Supplementary file 1 [file DataSheet1.pdf]

Supplementary information

Supplementary Materials

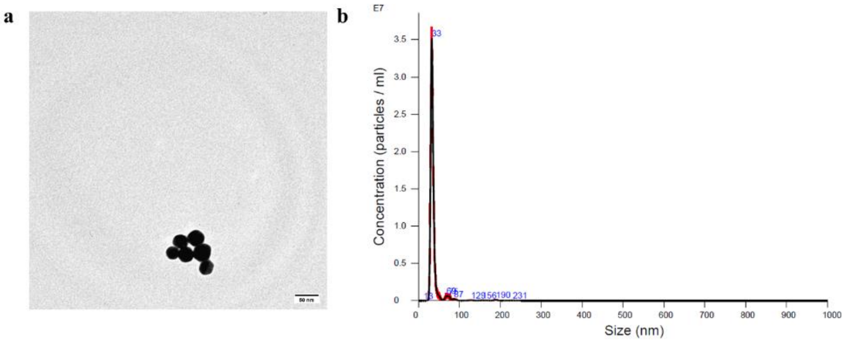

Figure S1. (a) TEM image of standard gold nanoparticles (scale bar = 50 nm). (b) Size distribution and concentration of gold nanoparticles determined by the nanoparticle tracking analysis (NTA).

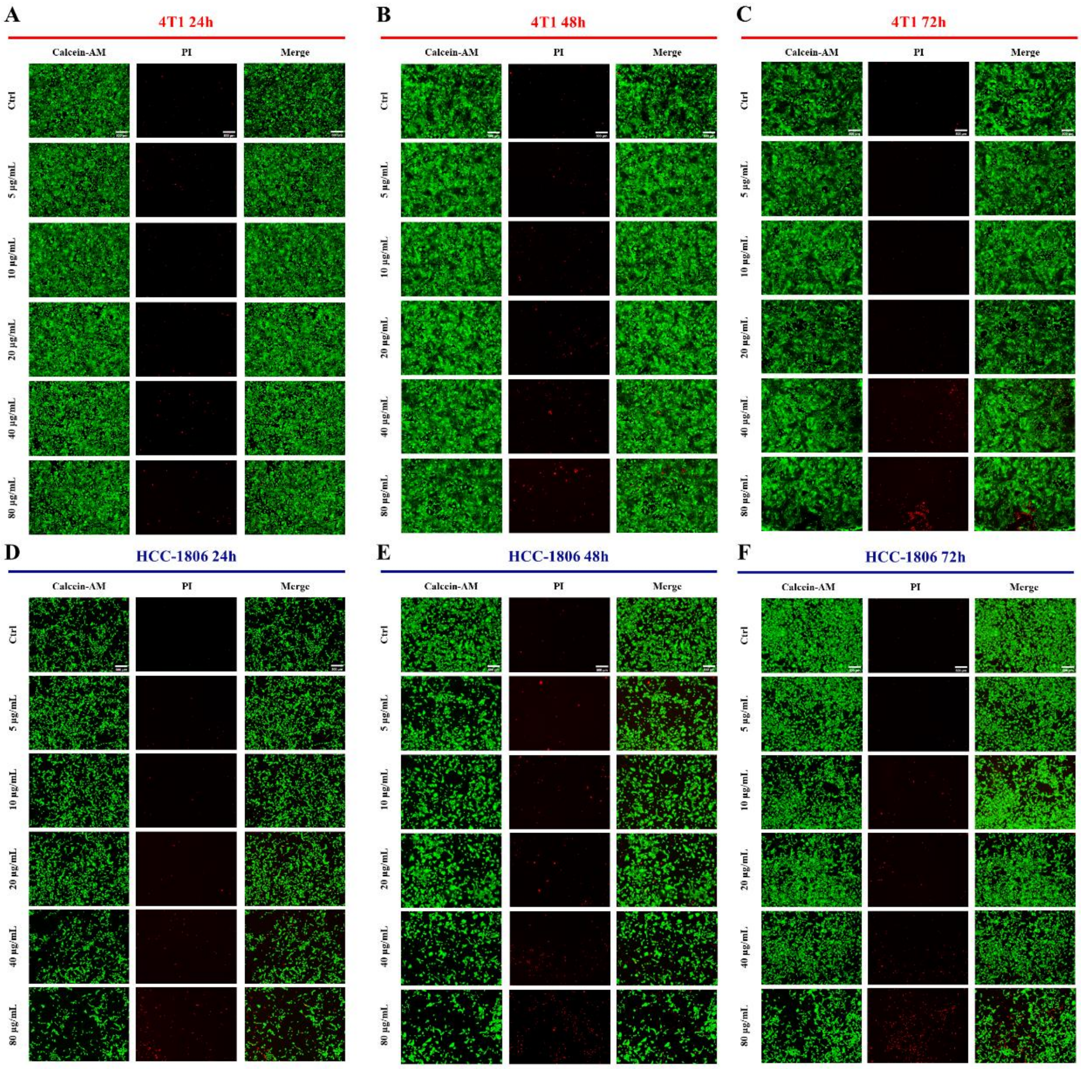

Figure S2. Effect of CLENs on the cell viability of 4T1 and HCC-1806 cells. Calcein-PI live-dead assay showing the effect of different concentrations of CLENs on cell viability. Representative fluorescence images of 4T1 (A-C) and HCC-1806 (D-F) cells received 5, 10, 20, 40 and 80 μg/mL CLENs for 24h, 48h and 72h (scale bar = 500 μm).

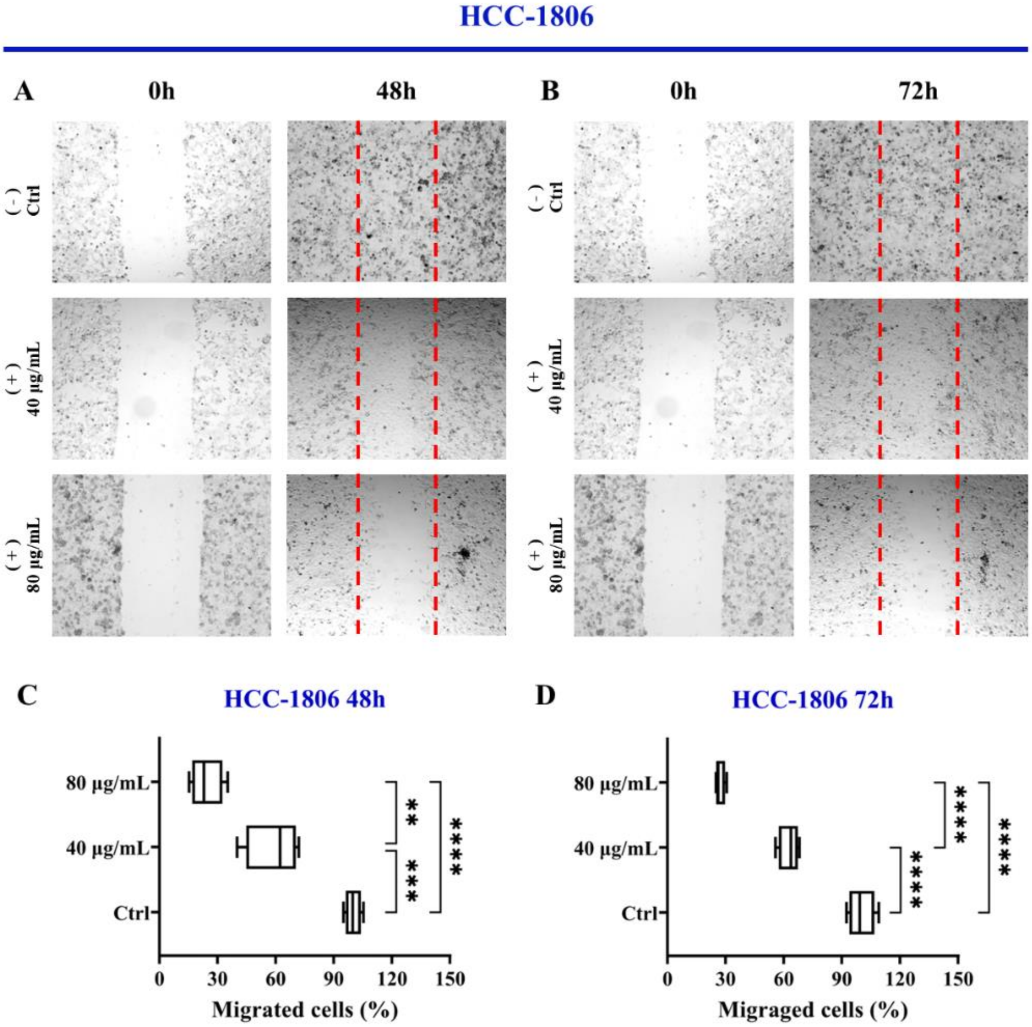

Figure S3. Wound healing assay performed in HCC-1806 cells scratched to cause a wound. Cells were incubated without or with 40 and 80 μg/mL CLENs. Representative images are shown the cell migration into wound area 48h (A) and 72h (B) post-wounding. Graphical representation of data from three independent experiments performed in triplicate. Bars, Mean +/- SEM. * p<0.05; ** p<0.01; One-way ANOVA followed by Tukey's multiple comparisons test.

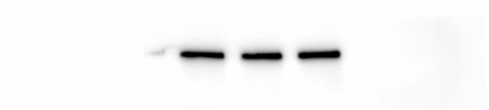

Figure S4. Original Western blot image of PI3K, samples of control (extracted proteins from untreated 4T1 cells), CLENs treatment for 72h cell lysate from three independent experiments. Samples were added in the following order: control, 40 μg/mL CLENs, 80 μg/mL CLENs.

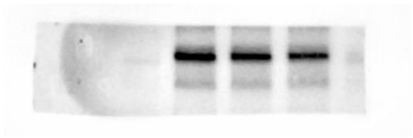

Figure S5. Original Western blot image of phosphorylated PI3K (p-PI3K), samples of control (extracted proteins from untreated 4T1 cells), CLENs treatment for 72h cell lysate from three independent experiments. Samples were added in the following order: control, 40 μg/mL CLENs, 80 μg/mL CLENs.

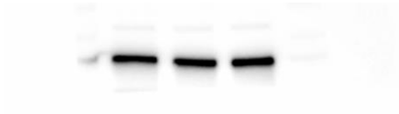

Figure S6. Original Western blot image of AKT, samples of control (extracted proteins from untreated 4T1 cells), CLENs treatment for 72h cell lysate from three independent experiments. Samples were added in the following order: control, 40 μg/mL CLENs, 80 μg/mL CLENs.

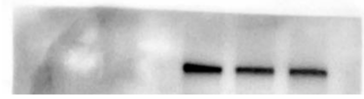

Figure S7. Original Western blot image of phosphorylated AKT (p-AKT), samples of control (extracted proteins from untreated 4T1 cells), CLENs treatment for 72h cell lysate from three independent experiments. Samples were added in the following order: control, 40 μg/mL CLENs, 80 μg/mL CLENs.

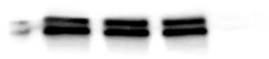

Figure S8. Original Western blot image of ERK, samples of control (extracted proteins from untreated 4T1 cells), CLENs treatment for 72h cell lysate from three independent experiments. Samples were added in the following order: control, 40 μg/mL CLENs, 80 μg/mL CLENs.

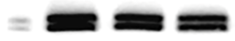

Figure S9. Original Western blot image of phosphorylated ERK (p-ERK), samples of control (extracted proteins from untreated 4T1 cells), CLENs treatment for 72h cell lysate from three independent experiments. Samples were added in the following order: control, 40 μg/mL CLENs, 80 μg/mL CLENs.

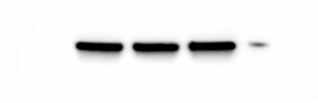

Figure S10. Original Western blot image of β-actin, samples of control (extracted proteins from untreated 4T1 cells), CLENs treatment for 72h cell lysate from three independent experiments. Samples were added in the following order: control, 40 μg/mL CLENs, 80 μg/mL CLENs.

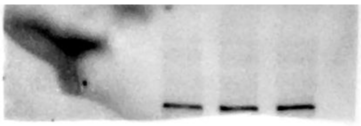

Figure S11. Original Western blot image of PI3K, samples of control (extracted proteins from untreated HCC-1806 cells), CLENs treatment for 72h cell lysate from three independent experiments. Samples were added in the following order: control, 40 μg/mL CLENs, 80 μg/mL CLENs.

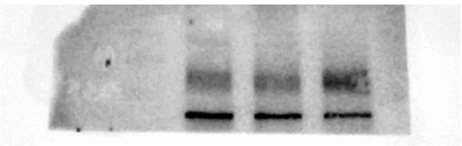

Figure S12. Original Western blot image of phosphorylated PI3K (p-PI3K), samples of control (extracted proteins from untreated HCC-1806 cells), CLENs treatment for 72h cell lysate from three independent experiments. Samples were added in the following order: control, 40 μg/mL CLENs, 80 μg/mL CLENs.

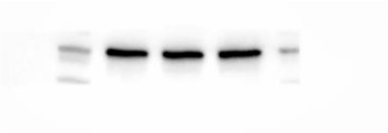

Figure S13. Original Western blot image of AKT, samples of control (extracted proteins from untreated HCC-1806 cells), CLENs treatment for 72h cell lysate from three independent experiments. Samples were added in the following order: control, 40 μg/mL CLENs, 80 μg/mL CLENs.

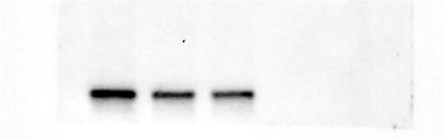

Figure S14. Original Western blot image of phosphorylated AKT (p-AKT), samples of control (extracted proteins from untreated HCC-1806 cells), CLENs treatment for 72h cell lysate from three independent experiments. Samples were added in the following order: control, 40 μg/mL CLENs, 80 μg/mL CLENs.

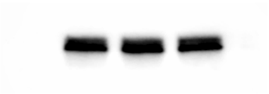

Figure S15. Original Western blot image of ERK, samples of control (extracted proteins from untreated HCC-1806 cells), CLENs treatment for 72h cell lysate from three independent experiments. Samples were added in the following order: control, 40 μg/mL CLENs, 80 μg/mL CLENs.

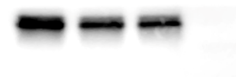

Figure S16. Original Western blot image of phosphorylated ERK (p-ERK), samples of control (extracted proteins from untreated HCC-1806 cells), CLENs treatment for 72h cell lysate from three independent experiments. Samples were added in the following order: control, 40 μg/mL CLENs, 80 μg/mL CLENs.

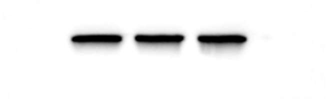

Figure S17. Original Western blot image of β-actin, samples of control (extracted proteins from untreated HCC-1806 cells), CLENs treatment for 72h cell lysate from three independent experiments. Samples were added in the following order: control, 40 μg/mL CLENs, 80 μg/mL CLENs.
